# Book review: A long-awaited book about spiders from the Asian part of Russia

**DOI:** 10.3897/zookeys.154.2339

**Published:** 2011-12-12

**Authors:** Mikhail M. Omelko, David Penney

**Affiliations:** 1 Far Eastern Federal University, Sukhanova, 8, Vladivostok 690950 Russia; 2 Gornotaezhnaya Station FEB RAS, Gornotaezhnoe Vil., Ussuriyski Dist., Primorski Krai 692533 Russia; 3 Faculty of Life Sciences, Michael Smith Bld, University of Manchester, M13 9PT, UK

The authors of the book are well known scientists in Russian arachnology. Yuri Marusik has written more than 250 articles and several monographs. As a result of his work the number of known species from this region has increased from 500 at the end of the 1970s to approximately 2000 at present. He has described more than 400 new species and genera as well as having compiled check-lists for the majority of sub-regions of the Asian part of Russia. Yuri Marusik has reported many new records for the region and sometimes for the entire country. For example, many of the following families he recorded were previously unknown: Leptonetidae, Theridiosomatidae, Mysmenidae, Ctenidae, Cybaeidae, Oonopidae and Nesticidae.

**Figure:**
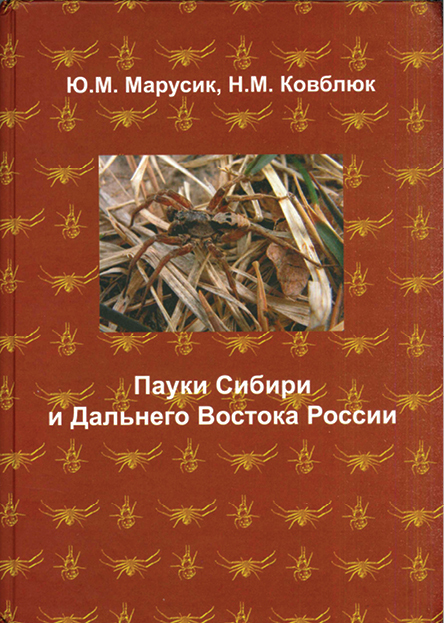


The second author – Nikolay Kovblyuk – works primarily in the Crimea (Ukraine) but has written a number of articles with Yuri Marusik or by himself concerning many taxa from Siberia and the Russian Far East. The book was edited by Professor B.R. Striganova of the Russian Academy of Sciences and is dedicated to Dr Kirill Eskov who was the first Russian researcher to make significant contributions to our knowledge on the spider fauna of this region.

Researchers beginning the study of spiders in Russia, especially in its Asian region immediately encountered the problem of an absence of identification guides and a dearth of other literature containing summarized data known to date. Sometimes even assigning collected spiders to family level was problematic (when the genus was known this task was easily accomplished using Platnick’s catalogue). Only one identification guide for spiders (covering a large area: the European part of the former USSR, and incorporating more than 1000 species) has been written in Russian, but this was published more than 40 years ago (Tyshchenko 1971). Since then spider classification has undergone major changes. The limits of many families have been revised, their number has almost doubled, many new taxa have been found and/or described. Moreover, Tyshchenko’s guide covered only the European part of the country, so it is not particularly useful for the identification of spiders in families which do not occur to the east of the Ural Mountains. Therefore, the publication of a book with identification keys and descriptions of families for this previously uncovered region was a necessary and long-awaited event.

The book consists of an abstract, the contents, 4 main chapters, 5 appendices and the index. The abstract and contents are provided both in Russian and English, but the remaining text is in Russian only. In the introduction (Chapter 1) the authors give basic information about the order, the region covered, some historical data, and comment on the aim of the book. The detailed historical review is subdivided into two parts. The first includes information about the study of spiders in the whole of Russia and the second is restricted to the Asian part of the country. This section discusses the contributions from all arachnologists (past and present) which have researched the spider fauna of this region.

Beyond doubt, chapter 2, which explains the methods pertaining to research on spiders, will be extremely useful for all beginners and even professional arachnologists. At the beginning of this chapter the authors give a list of the equipment necessary for collecting spiders. This is followed by descriptions of all the main collecting methods: by hand in different habitats, sifting the litter, pitfall trapping, sweep net sampling and so on. Information about the best time to collect spiders, with regard to different habitats and seasons is also provided. Most of this chapter is devoted to methods of processing collected spiders: how to examine them using a microscope, how to extract the epigyne and so forth. The procedures for drawing and photographing the copulatory organs are discussed over several pages. At the end the authors give recommendations as two which literature should be used and how to recognize the spiders.

This chapter also includes a number of pictures illustrating different types of equipment for collecting and studying material, examples of drawings and so on. It is known to the first author of this review, that most of these images were supposed to have been printed in colour, but in the final version of the book they are black-and-white. However, this does not affect the merits of this chapter. There are descriptions of some original methods for collecting spiders which have never been described in the literature before. For example, the authors write that some interesting spiders can be collected from water while standing within the water body itself (surface of the water should be at neck level). Also in this chapter the authors offer advice on the practicalities of studying spiders. They suggest the use of dishes with paraffin on the bottom in order to place a specimen or its copulatory organs in the proper position for taking pictures. Tiny glass beads (used in chromatography) are often used for this purpose in other countries, but they are not easy to obtain in Russia.

Chapter 3 covers the morphology and classification of spiders. One important aspect of this chapter are the keys for recognizing spider families that occur in Siberia and the Russian Far East. The authors provide five keys and a pictorial identification guide. Three of the keys are for all spider families (pictorial, dichotomous and multi-entry keys), one key is intended for araneoid families only and the last one is for families which are poorly recognized and utilizes easy characters such as eyes, spinnerets, legs and so forth. These keys certainly represent the most important part of the chapter and maybe even of the book as a whole, at least for non-arachnologists. Thanks to these keys anyone (student, arachnologist, amateur or entomologist) will be able to identify a spider to family level. Unfortunately there are no keys for genera. In our opinion it would be easy to make such keys at least for some of the families that are not so diverse in this region.

Chapter 4 is the most voluminous and occupies about half of the book. Here, descriptions of all families occurring in the Asian part of Russia are given as well as three additional families (Oecobiidae, Segestriidae and Zodariidae) that are currently unknown from this territory but may be expected to be found there in the future. All descriptions are made in a standard way similar to those in the books by Dippenaar-Schoeman and Jocqué (1997) and Jocqué and Dippenaar-Schoeman (2006). Each subsection includes diagnostic characters of a particular family, a list of taxa within a family, description, genera known, distribution, details on biology, collecting methods, taxonomy, ways of differentiation of species and opportunities for further investigations. The description of each family is more detailed than in the two books mentioned above and comprise a lot of drawings and photos (up to nearly 30 per family) to illustrate the copulatory organs, details of external morphology as well as living spiders in their natural habitat. The big merit is that all the photos are printed in colour. Unfortunately, the high quality of the original photos has been lost in part while printing, therefore some images have superfluous contrast or look irregular (e.g. figs on pages 121, 125, 177, 259 and some more), and many of the black-and-white drawings have a greenish tint. In several cases the arrangement of some pictures seems strange. For example, the photo of *Cheiracanthium* sp. (Cheiracanthiidae) follows the description of Leptonetidae (p. 167), photos of two crab-spiders (Thomisidae) are located after Mysmenidae, a photo of *Marpissa pulla* (Salticidae) occurs on p. 249 in the Theridiosomatidae section, and so on. According to information from the senior author of the book this arrangement was made in order to occupy empty spaces, in cases where photos of spiders of a particular family were absent. However, this is going to be misleading because, taking the last example, the specific epithet *pulla* is not listed in the index and *Marpissa* is listed, but only as appearing on pages 224 and 316; thus the index provides no reference to the photograph which occurs out of systematic context within this chapter. One genus (*Diphya*, Tetragnathidae) and one species (*Callobius hokkaidensis*, Amaurobiidae) are reported from Russia for the first time. Each description ends with the section “Prospects for further investigations”. Here, useful information about how many described species can be found in the region, which scientist is studying this particular family in Russia and so on is given.

In spite of these small flaws the chapter is rather interesting and provides a wealth of information. In addition to recent families, the authors provide brief information about fossil spiders, including the two extinct families Juraraneidae and Lagonomegopidae, and the extant Mecicobothriidae, which have been found in the Asian part of Russia. For these families no illustrations are provided, which is a shame because this would have made this chapter a little more substantial, rather than appearing as a single, incomplete page tagged on at the end. The authors also overlooked the publication of Selden (2010) which described several well preserved specimens of Theridiosomatidae from Transbaikalia.

There are several appendices in the book. The first concerns the etymology of spider genus names and is rather interesting. Some of these etymologies are derived from Cameron (2005), but many of the names of taxa that do not occur in the Nearctic are original. In addition, there are lists of Russian-speaking arachnologists (including address and group of interest), web sites useful for recognizing spiders, books and a glossary of terms. The book ends with a comprehensive index to both genera and species.

In terms of the technical production, we have critiqued the quality of some of the images above. The paper has a nice, glossy finish, but unfortunately is a little too thin, resulting in show through from the opposite side of the page. This is not a big problem, but it does generate an upleasing finish to what was obviously a lot of hard work. The contents do not always match up with the pages on which the sections supposedly start. For example, the fossil families are indicated to start on page 278, but they actually occur on page 279. For some reason in the design process the decision was made to omit page numbers from pages which start a new section. This is rather bizarre because the contents should direct the reader to a numbered page at the start of a specific section. This has resulted in 52 pages without numbers, which equates to around 15% of all pages.

Despite the few small drawbacks mentioned above, publication of this book is an extremely important event for Russian arachnology, and it will doubtless form a benchmark reference work for the foreseeable future. In our opinion the book will be of great interest among Russian-speaking readers. It is possible that publication of this book will stimulate investigations in Russia and adjacent countries. For specialists who cannot read Russian text, the book will be useful because of the numerous, clear pictures (there are more than 600 illustrations, both in colour and black-and-white), the majority of which have not been published before. Thus, we recommend this book for all Russian-based zoological libraries, but also to other libraries of individuals and institutions that may have an arachnological research interest in this region. The book will also be of use to the Russian-speaking layperson with an interest in the natural history of spiders.
